# Biopsy-Proven Intestinal Failure-Associated Liver Disease in Pediatric Short Bowel Syndrome: Histopathological Findings and Clinical Outcomes

**DOI:** 10.3390/diagnostics16142205

**Published:** 2026-07-15

**Authors:** Miray Karakoyun, Doğan Barut, Bora Kunay, Eylem Tazegül Çokgezer, Claudia Andrea Gomez Gonzalez, Ülgen Çeltik, Ezgi Kıran Taşcı, Deniz Nart, Funda Yılmaz, Ahmet Çelik, Funda Çetin

**Affiliations:** 1 Department of Pediatric Gastroenterology, Hepatology and Nutrition, Medical School, Ege University; Izmir 35100, Turkey; miray.karakoyun@ege.edu.tr (M.K.); borakunay@gmail.com (B.K.); eylemtazegul@gmail.com (E.T.Ç.); ezgikiran@gmail.com (E.K.T.); fndzgnc@gmail.com (F.Ç.); 2 Department of Pathology, Medical School, Ege University; Izmir 35100, Turkey; clau.gomezg@gmail.com (C.A.G.G.); deniz.nart@ege.edu.tr (D.N.); funda.yilmaz@ege.edu.tr (F.Y.); 3 Department of Pediatric Surgery, Medical School, Ege University, Izmir 35100, Turkey; ulgenceltik1988@gmail.com (Ü.Ç.); celikahmet969@gmail.com (A.Ç.)

**Keywords:** intestinal failure-associated liver disease, parenteral nutrition, short-bowel syndrome

## Abstract

**Background/Objectives**: Intestinal failure-associated liver disease (IFALD), characterized by steatosis and/or cholestasis, may develop in patients receiving long-term parenteral nutrition (PN) for chronic intestinal failure. This study aimed to evaluate the histopathological features and clinical outcomes of IFALD in children with short bowel syndrome (SBS) enrolled in an Intestinal Rehabilitation Program (IRP) between 2015 and 2022. **Methods**: Data from 72 children with SBS requiring PN were retrospectively reviewed. Liver biopsy specimens were obtained during bowel-lengthening procedures or surgical exploration. Histopathological findings and clinical outcomes were analyzed. Active IFALD (phase 1) (cholestasis/inflammation; see below for full description) is diagnosed when serum direct/conjugated bilirubin is >1.0–2.0 mg/dL and >20% of total serum bilirubin in infants and children who have received PN for at least 14 days, and in whom other causes of cholestasis have been excluded. Liver histology may show cellular and canalicular cholestasis, bile ductular reaction, and portal bile plugs similar to those observed in extrahepatic biliary obstruction, variable portal inflammation, steatosis, and periportal fibrosis of all stages, as well as prominent liver macrophages. Chronic IFALD is the persistent form of intestinal failure-associated liver disease characterized by established hepatic fibrosis and/or cirrhosis resulting from prolonged intestinal failure and parenteral nutrition exposure, with or without persistent biochemical evidence of cholestasis. **Results**: Sixteen patients underwent diagnostic liver biopsy at a median age of 14 months (range, 3–62 months). Histopathological evaluation demonstrated cholestasis in 13 patients (81.3%) and steatosis in 2 patients (12.5%). Among patients with cholestasis, intracellular and canalicular patterns were observed in 10 (62.5%) and 6 (37.5%) patients, respectively. Fibrosis was identified in 5 patients (31.3%), including stage 1 in 3, stage 2 in 1, and stage 3 (bridging) in 1. Active IFALD and chronic IFALD were detected in 13 and 5 patients, respectively. One patient died due to IFALD-related end-stage liver disease. **Conclusions**: IFALD remains a significant and potentially fatal complication in children with SBS receiving long-term PN. Histopathological evidence of liver injury may precede biochemical abnormalities, emphasizing the importance of early surveillance, optimization of PN strategies, and timely achievement of enteral autonomy.

## 1. Introduction

Intestinal failure-associated liver disease (IFALD) remains a complex and multifactorial complication in patients with short bowel syndrome (SBS) and chronic intestinal failure (IF). IFALD encompasses a spectrum of hepatic abnormalities, including cholestasis, inflammation, fibrosis, and steatosis. While cholestasis, inflammation, and fibrosis are more commonly observed in neonates and infants, steatosis tends to predominate in older children and adults with IFALD [1,2,3].

The pathogenesis of IFALD is closely linked to the reduced functional intestinal surface area in patients with short bowel syndrome (SBS), leading to impaired fluid and nutrient absorption and compromised growth. Neonatal SBS most commonly develops following extensive bowel resection due to necrotizing enterocolitis or congenital intestinal anomalies. Severe intestinal dysmotility disorders, such as Hirschsprung disease requiring high jejunostomy, may also contribute to the development of SBS and subsequent IFALD [4,5].

Parenteral nutrition (PN) remains a life-saving therapy for children with SBS; however, prolonged PN exposure predisposes patients to PN-associated cholestasis and IFALD. Despite advances in multidisciplinary intestinal rehabilitation and nutritional management, IFALD continues to represent a significant source of morbidity and mortality in children with IF [6,7].

Several clinical risk factors have been associated with IFALD, including prematurity, low birth weight, recurrent sepsis, lack of enteral feeding, and prolonged administration of soybean oil-based lipid emulsions rich in ω-6 fatty acids and plant sterols. These factors contribute to the multifactorial pathogenesis of IFALD [8,9,10,11,12,13].

IFALD is typically diagnosed based on cholestatic liver biochemical abnormalities in patients receiving prolonged PN after exclusion of other causes of liver disease. In addition to routine liver biochemistry, noninvasive methods such as liver stiffness measurements have shown potential in monitoring IFALD; however, their diagnostic accuracy has rarely been validated against liver histopathology [14,15].

The acute phase of IFALD is predominantly characterized by cholestasis and inflammation, whereas fibrosis and steatosis become more prominent in the chronic phase. Cholestasis is often reversible following optimization of PN strategies and restoration of enteral nutrition. In contrast, fibrosis and steatosis may persist for years, even after discontinuation of PN. Although steatosis may develop during early infancy, its role in the progression of chronic IFALD remains insufficiently understood [3,16,17,18].

Active IFALD (phase 1) (cholestasis/inflammation; see below for full description) is diagnosed when serum direct/conjugated bilirubin is >1.0–2.0 mg/dL and >20% of total serum bilirubin in infants and children who have received PN for at least 14 days, and in whom other causes of cholestasis have been excluded. Liver histology may show cellular and canalicular cholestasis, bile ductular reaction, and portal bile plugs similar to those observed in extrahepatic biliary obstruction, variable portal inflammation, steatosis, and periportal fibrosis of all stages, as well as prominent liver macrophages.

Chronic IFALD (phase2) (steatosis/fibrosis) does not have any generally accepted diagnostic criteria; thus, diagnosis may require liver biopsy until less-invasive biomarkers progressing to biliary cirrhosis, steatohepatitis, and gallbladder disease, in patients who receive prolonged courses of PN when other causes of liver injury and disease have been excluded [3].

Chronic IFALD is the persistent form of intestinal failure-associated liver disease characterized by established hepatic fibrosis and/or cirrhosis resulting from prolonged intestinal failure and parenteral nutrition exposure, with or without persistent biochemical evidence of cholestasis.

Approximately two-thirds of neonates receiving prolonged PN develop cholestasis, and 20–30% of children with long-term PN dependence develop clinically evident IFALD. Furthermore, advanced liver fibrosis is identified in nearly 60% of patients evaluated for transplantation [4,19,20].

Enteral autonomy was defined as normal weight gain with full enteral provisions and permanent cessation of parenteral nutrition [21].

Although initial cholestasis may resolve after optimization of PN and achievement of enteral autonomy, persistent abnormalities in liver histology and biochemistry remain common among long-term survivors with IF. However, the long-term progression of IFALD and the most reliable methods for disease monitoring remain unclear [3,6,22,23,24,25].

Therefore, this study aimed to evaluate the histopathological features and progression of IFALD in children with IF receiving long-term PN at a tertiary referral center. Serial liver biopsy findings were analyzed by IFALD activity stage to assess disease severity, progression, and clinical outcomes.

## 2. Materials and Methods

### 2.1. Study Design, Patients, and Data Collection

Clinical outcomes of children with SBS who developed intestinal failure-associated liver disease (IFALD) within the Intestinal Rehabilitation Program (IRP) were retrospectively analyzed. This single-center follow-up study was based on prospectively collected data from pediatric patients with intestinal failure (IF) managed at a tertiary pediatric referral center in Türkiye between 2015 and 2022. The study was approved by the Ege University Committee for Clinical Research Ethics (decision no. 24-4T/1, dated 4 April 2024). Of 72 eligible patients, 16 consecutive patients who underwent at least 1 liver biopsy to evaluate IF-associated liver histopathology were included. Clinical and demographic data, including sex, gestational age, PN duration and composition, PN lipid type, sepsis episodes, liver histopathology, survival, and cause of death, were obtained from a prospective institutional database. Dates of initiation and discontinuation of PN were recorded. Laboratory parameters, PN volume, and macronutrient composition at baseline and at approximately 1-, 3-, 6-, 9-, and 12-month follow-up intervals, along with liver biopsy findings, were extracted from patient records. Our study includes patients with short bowel syndrome who underwent liver biopsy between 2015 and 2022. Samples from patients who had liver biopsies during surgery are reported in approximately one week. We retrospectively collected those reports and blood test results.

Patients were classified according to histopathological disease activity stages based on diagnostic and follow-up liver biopsy findings. Alternative causes of liver disease were excluded in all patients. Median values are reported.

Since 2015, patients with IF have been managed according to a standardized multidisciplinary intestinal rehabilitation protocol, including optimized catheter care, balanced PN administration, early enteral feeding, cycling PN when feasible, and fish oil-based lipid therapy in selected patients with cholestasis or severe SBS. Most patients received a combination of fish oil-based and mixed lipid emulsions. Patients referred from other institutions may have received different PN lipid strategies before admission to our center.

### 2.2. Laboratory Assessment and Definitions

Blood samples obtained at the time of liver biopsy were analyzed for aspartate aminotransferase (AST), alanine aminotransferase (ALT), bile acids, gamma-glutamyl transferase (GGT), and conjugated bilirubin in the hospital laboratory.

Cholestasis defined as ALT, ALP, and/or GGT levels exceeding 1.5 times the upper limit of the reference range for at least 6 weeks, with total bilirubin levels of 50–100 μmol/L, of which ≥50% was conjugated bilirubin or total bilirubin > 100 μmol/L [26,27].

Type 1 IFALD was defined as ALT, ALP, and/or GGT levels exceeding 1.5 times the upper limit of the reference range for at least 6 weeks, with total bilirubin < 50 μmol/L. Type 2 IFALD was defined as type 1 IFALD with total bilirubin levels of 50–100 μmol/L, of which ≥50% was conjugated bilirubin. Type 3 IFALD was defined as end-stage liver disease with clinical manifestations and total bilirubin > 100 μmol/L [27].

Sepsis was defined as a positive peripheral or central venous blood culture in a symptomatic child without another identifiable focus of infection. Blood cultures were routinely obtained before initiation of antibiotic therapy.

### 2.3. Liver Biopsy and Histopathological Evaluation

Diagnostic core needle liver biopsies were obtained under general anesthesia from patients with short bowel syndrome (SBS), either during routine follow-up endoscopic procedures or at the time of laparotomy. Follow-up biopsies were performed in patients considered to be at high risk for progression of liver disease. Core needle biopsies obtained during laparotomy were performed by pediatric surgeons.

All biopsy specimens were independently evaluated by two experienced pediatric pathologists. Histopathological assessment included routine hematoxylin and eosin staining, as well as Masson trichrome, iron, and reticulin staining. Steatosis was graded according to the proportion of affected hepatocytes as follows: grade 0, absent; grade 1, <33%; grade 2, 33–50%; and grade 3, >50%. Portal inflammation was graded as absent (0), minimal (1), mild (2), moderate (3), or extensive (4). Fibrosis was staged according to the METAVIR scoring system as stages 1 (portal fibrosis), 2 (portal fibrosis with few septa), 3 (bridging fibrosis), and 4 (cirrhosis).

Cholestasis was graded based on the highest degree of intracellular, canalicular, or ductular cholestasis observed: grade 0, absent; grade 1, minimal; grade 2, marked; and grade 4, prominent, as previously described [16].

### 2.4. Statistical Analysis

Nonparametric methods were used for data analysis, and logistic regression analysis was performed to identify determinants of IFALD. Descriptive statistics are presented as frequencies and medians (interquartile ranges [IQRs]). The Kruskal–Wallis test or Pearson’s chi-square test was used for comparisons among multiple groups, whereas the Mann–Whitney U test or Fisher’s exact test was used for comparisons between two groups. All statistical analyses were performed using SPSS version 18 (SPSS Inc., Chicago, IL, USA). Odds ratios (ORs) with 95% confidence intervals (CIs) were calculated using simple logistic regression analysis. A *p* value of ≤0.05 was considered statistically significant.

## 3. Results

### 3.1. Patient Characteristics

**Figure 1:**
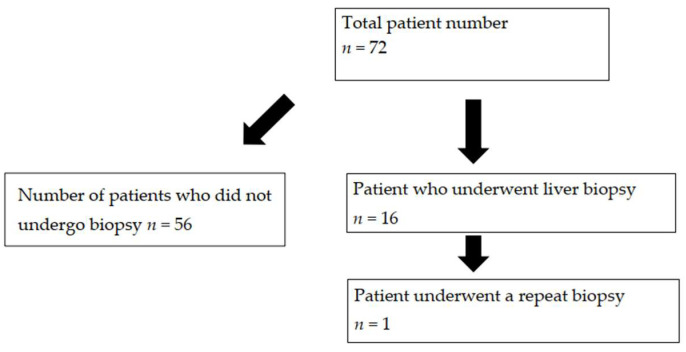
Patient flow diagram.

Among 72 children with SBS requiring PN enrolled in the IRP, 16 who underwent liver biopsy to evaluate IF-associated liver histopathology were included in the analysis. Baseline demographic and clinical characteristics of these patients are summarized in Table 1. Sixteen patients who underwent liver biopsy are those who underwent bowel lengthening surgery or surgical exploration. Follow-up biopsies were performed in one patients considered to be at high risk for progression of liver disease (Figure 1).

The median age at liver biopsy was 14 months (3–62 months), and 56.3% of the patients were born prematurely. Intestinal atresia was the most common etiology of SBS (50%), followed by necrotizing enterocolitis (25%). The median residual small bowel length was 45.8 cm, and the ileocecal valve was preserved in 68.7% of patients. Full colon preservation was present in 68.7% of cases. Median ALT, GGT, and total bilirubin levels at referral were 108 U/L, 141 U/L, and 2.5 mg/dL, respectively, while biochemical cholestasis was detected in 50% of the patients.

Clinical, biochemical, and nutritional characteristics at the time of liver biopsy are summarized in Table 2. The median interval between admission and liver biopsy was 8.5 months, while the median duration of PN was 21.5 months. Median ALT, GGT, and total bilirubin levels at biopsy were 70 U/L, 72 U/L, and 1.19 mg/dL, respectively. Biochemical cholestasis was present in 31.2% of patients. The median proportion of total energy provided by PN was 80%, and the median lipid supply was 2 g/kg/day. Most patients (87.5%) experienced septic episodes during follow-up, with a median of 3.4 septic episodes per patient.

### 3.2. Histopathological Findings

**Figure 2:**
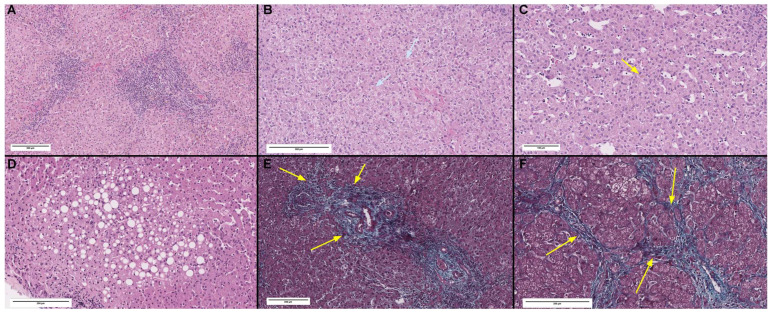
Histopathological findings of active and chronic IFALD in liver biopsies. (**A**) Portal inflammation: an inflammatory cell infiltrate is observed within the portal tracts (active IFALD). (**B**) Intracellular cholestasis (blue arrow; active IFALD). (**C**) Intracanalicular cholestasis (yellow arrow; active IFALD). (**D**) Steatosis: macrovesicular steatosis is visible within the liver parenchyma (chronic IFALD). (**E**) Portal fibrosis expansion (demarcated by yellow arrows; chronic IFALD; Masson’s trichrome staining). (**F**) Bridging fibrosis (portal–portal bridging indicated by yellow arrows; chronic IFALD; Masson’s trichrome staining). Scale bars are included in each panel.

Histopathological findings of liver biopsies are summarized in Table 3, and representative histopathological images are presented in Figure 2. Active IFALD, characterized by cholestasis and/or portal inflammation, was identified in 93.8% of patients, whereas chronic IFALD, including fibrosis and/or steatosis, was identified in 37.5% of patients. Histopathological cholestasis was observed in 81.3% of patients, with intracellular and canalicular cholestatic patterns detected in 62.5% and 37.5% of cases, respectively. Portal inflammation was present in 37.5% of patients, and fibrosis in 31.2%, including 1 patient with bridging fibrosis. Steatosis was detected in 12.5% of patients. One patient developed IFALD-related end-stage liver disease and died during follow-up.

Active IFALD, characterized by cholestasis and/or portal inflammation, was identified in 93.8% of patients, whereas chronic IFALD, including fibrosis and/or steatosis, was identified in 37.5%. Histopathological cholestasis was observed in 81.3% of patients, with intracellular and canalicular cholestatic patterns detected in 62.5% and 37.5% of cases, respectively. Portal inflammation was present in 37.5% of patients, and fibrosis in 31.2%, including 1 patient with bridging fibrosis. Steatosis was detected in 12.5% of patients. One patient developed IFALD-related end-stage liver disease and died during follow-up.

### 3.3. Clinical Characteristics According to Histopathological IFALD Phenotypes

Clinical and nutritional characteristics according to histopathological IFALD phenotypes are presented in Table 4. Active and chronic IFALD phenotypes were not mutually exclusive. Among the 16 patients, 10 demonstrated isolated active IFALD, 1 demonstrated isolated chronic IFALD, and 5 exhibited overlapping active and chronic histopathological features.

Prematurity was observed in 60% of patients with active IFALD and in 83% of those with chronic IFALD. The median duration of PN was 11 months in patients with active IFALD and 18 months in those with chronic IFALD. Septic episodes occurred in 86% and 83% of patients with active and chronic IFALD, respectively. Enteral autonomy was achieved in 40% of patients with active IFALD and in 83% of patients with chronic IFALD.

Among patients with chronic IFALD, preservation of the ileocecal valve and full colon was observed in 33% and 50% of patients, respectively.

### 3.4. Enteral Autonomy and Mortality

Enteral autonomy was achieved in 25% (*n* = 2) of patients with laboratory-defined IFALD and in 37.5% (*n* = 6) of those with biopsy-proven IFALD, whereas long-term PN dependency persisted in 75% (*n* = 6) and 62.5% (*n* = 10) of patients, respectively. One patient with biopsy-proven IFALD died due to IFALD-related end-stage liver disease during follow-up (Table 5).

## 4. Discussion

This study provides a comprehensive histopathological evaluation of IFALD in children with SBS receiving long-term parenteral nutrition within a multidisciplinary intestinal rehabilitation program. By integrating histopathological, biochemical, nutritional, and clinical outcome data, the study aimed to characterize active and chronic IFALD phenotypes and evaluate their association with PN dependency, septic episodes, enteral autonomy, and mortality.

In the present cohort, histopathological evidence of IFALD was identified in the majority of patients undergoing liver biopsy. Active cholestatic/inflammatory IFALD, characterized by cholestasis and/or portal inflammation, represented the predominant phenotype and was detected in 93.8% of patients, whereas chronic IFALD findings, including fibrosis and/or steatosis, were identified in 37.5% of patients. Histopathological cholestasis was the most frequent finding, while fibrosis and steatosis were less common. One patient progressed to IFALD-related end-stage liver disease and died during follow-up.

An important finding of this study is that significant histopathological liver injury was detected even in some patients without marked biochemical cholestasis. Although biochemical cholestasis was present in only a subset of patients at the time of liver biopsy, biopsy-proven IFALD was identified in nearly all patients. These findings suggest that histopathological liver injury may precede overt biochemical abnormalities in children with intestinal failure receiving prolonged PN. Previous studies have similarly demonstrated that biochemical markers alone may underestimate the severity of IFALD and that persistent histopathological abnormalities may remain despite improvement in laboratory findings [3,16,23].

The predominance of active cholestatic/inflammatory IFALD observed in our cohort is clinically important because cholestatic liver injury represents the potentially reversible phase of IFALD and may respond to optimization of intestinal rehabilitation strategies. Patients with active IFALD commonly demonstrated prolonged PN dependency, recurrent septic episodes, and persistent intestinal failure. Recurrent sepsis is a well-recognized contributor to IFALD progression through repeated inflammatory insults, bacterial translocation, and catheter-related bloodstream infections [8,9,10,11,12,13]. Similarly, prolonged PN exposure and severe intestinal loss remain among the major determinants of cholestatic liver injury in pediatric SBS.

The comparatively low incidence of advanced fibrosis and steatosis in our cohort may reflect the benefits of early multidisciplinary intestinal rehabilitation and modern PN strategies. Since 2015, patients in our center have been managed using standardized intestinal rehabilitation protocols, including early enteral feeding, catheter care optimization, cycling PN, and fish oil-based lipid therapy in selected patients with cholestasis or severe SBS. Previous studies have demonstrated that reducing phytosterol exposure and increasing anti-inflammatory lipid components through fish oil-based emulsions may improve cholestatic liver disease and reduce progression to advanced IFALD [9,28,29,30,31,32].

Most patients in our cohort remained dependent on long-term PN and failed to achieve enteral autonomy. Recurrent septic episodes and cholestatic liver injury may partly explain the relatively low enteral autonomy rate observed in this study, as both factors negatively affect intestinal adaptation and long-term nutritional outcomes. Early initiation and advancement of enteral nutrition remain essential to promote intestinal adaptation and facilitate weaning from PN [13,33,34].

Our findings also support the potential role of liver biopsy in selected children with prolonged PN dependency and ongoing clinical suspicion of IFALD, even when biochemical abnormalities are mild or absent. Histopathological evaluation may provide important information regarding disease activity and chronicity that cannot be fully captured by laboratory parameters alone. Increased GGT levels have previously been associated with better response to fish oil-based lipid therapy in cholestatic IFALD, possibly reflecting an active inflammatory phenotype that remains responsive to therapeutic intervention [23].

This study has several limitations. First, only a subset of eligible patients underwent liver biopsy, which may have introduced selection bias toward patients with more severe intestinal failure and prolonged PN dependency. Second, the single-center design and relatively small sample size may limit the generalizability of the findings. In addition, noninvasive diagnostic tools, including liver stiffness measurements and imaging-based fibrosis assessment, were not systematically evaluated or compared with histopathological findings. Furthermore, serial follow-up biopsies were not available for all patients, limiting evaluation of long-term histopathological progression.

Despite these limitations, the present study has important strengths. To our knowledge, this is one of the limited pediatric SBS cohorts evaluated with biopsy-proven IFALD during long-term follow-up within a standardized multidisciplinary intestinal rehabilitation program. The study provides detailed histopathological characterization of active and chronic IFALD phenotypes and demonstrates that clinically significant liver injury may be present despite relatively mild biochemical abnormalities. The integration of histopathological, biochemical, nutritional, and clinical outcome data provides clinically relevant insight into the natural history and monitoring of IFALD in children with SBS.

The management of intestinal failure-associated liver disease (IFALD) in pediatric short bowel syndrome is evolving toward earlier diagnosis, individualized risk stratification, and multidisciplinary care. Although liver biopsy remains the reference standard for assessing hepatic fibrosis and architectural changes, its invasive nature limits its routine use, particularly for longitudinal monitoring. Future studies should focus on validating reliable non-invasive biomarkers and imaging modalities capable of accurately detecting fibrosis, steatosis, and cholestatic injury in children with intestinal failure.

Although liver biopsy provides definitive histopathological assessment, several alternative approaches have increasing clinical relevance. Serum-based fibrosis indices, including pediatric adaptations of established fibrosis scores, are attractive because they are inexpensive and widely available; however, their performance in pediatric IFALD remains inconsistent and requires disease-specific validation.

Non-invasive imaging techniques, particularly transient elastography and magnetic resonance elastography, have shown promise for estimating liver stiffness and monitoring fibrosis over time. Nevertheless, liver stiffness measurements may be influenced by cholestasis, inflammation, congestion, and patient-related factors, limiting their specificity in children with intestinal failure. These techniques should therefore be interpreted alongside clinical and biochemical findings rather than replacing histological evaluation in selected patients.

From a therapeutic perspective, optimization of intestinal rehabilitation remains the cornerstone of clinical management. Early advancement of enteral nutrition, minimization of parenteral nutrition duration whenever feasible, use of modern intravenous lipid emulsions, prevention of catheter-related bloodstream infections, and coordinated multidisciplinary follow-up have demonstrated meaningful clinical benefit. Pharmacologic interventions, including glucagon-like peptide-2 analogues such as teduglutide, may reduce parenteral nutrition dependence in selected patients and thereby indirectly decrease the risk of IFALD, although further studies are needed to clarify their long-term effects on hepatic histopathology.

## 5. Conclusions

Histopathological IFALD was identified in the majority of children with SBS receiving long-term PN, with active cholestatic/inflammatory changes representing the predominant phenotype. Significant liver injury was detected even in some patients without marked biochemical abnormalities, suggesting that histopathological changes may precede overt laboratory evidence of liver disease. Recurrent sepsis, prolonged PN dependency, and failure to achieve enteral autonomy remained common among affected patients. These findings emphasize the importance of careful long-term monitoring and multidisciplinary intestinal rehabilitation strategies in children with intestinal failure at risk for IFALD.

## Figures and Tables

**Table 1:** Patient demographics and baseline characteristics.

Characteristics	N = 16
Gender, *n* (%)	
Male	13 (81.3)
Female	3 (18.7)
Age, months, median (min–max)	14 (3–62)
Prematurity, *n* (%)	9 (56.3)
Etiology of short bowel syndrome, *n* (%)	
Intestinal atresia	8 (50.0)
Necrotizing enterocolitis	4 (25.0)
Motility disorders	3 (18.7)
Mucosal diseases	1 (6.2)
Small bowel, cm, median (min–max)	45.8 (6–110)
Ileocecal valve preserved (*n*, %)	11 (68.7)
Hemi colon (*n*,%)	5 (31.2)
Full Colon (*n*,%)	11 (68.7)
ALT, median (min–max) (U/L)	108 (22–369)
GGT, median (min–max) (U/L)	141 (15–524)
Total bilirubin, (min–max) (mg/dL)	2.5 (0.3–9.9)
Cholestasis (*n*, %)	8 (50)

Data are expressed as median (minimum–maximum) or *n* (%). ALT, alanine aminotransferase; GGT, gamma-glutamyl transferase.

**Table 2:** Clinical and nutritional characteristics at the time of liver biopsy.

Patients	N = 16
Time from admission to liver biopsy (months, min–max)	8.5 (1–59 years)
ALT, median (min–max) (U/L)	70 (11–197)
GGT, median (min–max) (U/L)	72 (8–202)
Total bilirubin, median (min–max) (mg/dL)	1.19 (0.3–3.7)
Cholestasis, *n* (%)	5 (31.2)
Energy provided by PN, % of total energy (min–max)	80 (50–100)
Lipid supply in PN, median (min–max) (gr/kg)	2 (1.5–3.0)
Duration of PN, median (min–max) (months)	21.5 (4–120)
Patients with septic episodes, *n* (%)	14 (87.5)
Number of septic episodes per patient, median (min–max)	3.4 (0–12)

Data are expressed as median (minimum–maximum) or *n* (%). ALT, alanine aminotransferase; GGT, gamma-glutamyl transferase; PN, parenteral nutrition.

**Table 3:** Histopathological findings of liver biopsies in children with IFALD.

Patients	N = 16
Steatosis (*n*, %)	2 (12.5)
Cholestasis (*n*, %)	13 (81.25)
Intracellular cholestatic pattern	10 (62.5)
Canalicular cholestasis	6 (37.5)
Portal inflammation (*n*, %)	6 (37.5)
Fibrosis (*n*, %)	5 (31.2)
Periportal fibrotic expansion (Stage 1)	3 (18.7)
Periportal septal expansion (Stage 2)	1 (6.2)
Bridging fibrosis (Stage 3)	1 (6.2)
Iron in hepatocytes (*n*, %)	
Stage 0	7 (43.8)
Stage 1	3 (18.7)
Stage 2	5 (31.2)
Stage 3	1 (6.2)

**Table 4:** Predicting histopathological stages in diagnostic liver biopsy for IFALD.

	Active IFALD	Chronic IFALD	*p* Value Active IFALD, Chronic IFALD
Patients (*n*,%)	15 (93.75)	6 (37.5)	
Prematurity (*n*,%)	9 (60)	5 (83)	0.438–0.121
Short bowel group			
≤20 cm (*n*,%)	3 (20)	2 (33)	0.587–0.418
20–40 cm (*n*,%)	7 (46)	3 (50)	
≥40 cm (*n*,%)	5 (33)	1 (17)	
ICV preserved (*n*,%)	11 (73)	2 (33)	0.313–0.036
Hemi colon (*n*,%)	5 (33)	3 (50)	0.687–0.242
Full Colon (*n*,%)	10 (66)	3 (50)	
Laboratory cholestasis (*n*,%)	5 (33)	2 (33)	0.687–0.654
Septic episodes (*n*,%)	13 (86)	5 (83)	0.875–0.625
Total number/patient, *n*	4 (0–12)	6 (0–12)	0.042–0.200
Duration of PN(months)	11 (4–60)	18 (4–120)	0.141–0.384
Enteral autonomy (*n*,%)			
Positive	6 (40)	5 (83)	0.625–0.215
Negative	9 (60)	1 (17)	

**Table 5:** IFALD, enteral autonomy/mortality relationship.

	Laboratory IFALD (*n*:8, %)	Biopsy IFALD (*n*:16, %)	*p*
Enteral autonomy			
Positive	2 (25)	6 (37.5)	≥0.05
Negative	6 (75)	10 (62.5)	(0.608)
Mortality	-	1	≥0.05
			(0.375)

## Data Availability

The data are not publicly available due to privacy and ethical restrictions.

## Abbreviations

ALT	Alanine aminotransferase
AST	Aspartate aminotransferase
GGT	Gamma-glutamyl transferase
IF	Intestinal failure
IFALD	Intestinal failure-associated liver disease
IRP	Intestinal rehabilitation program
PN	Parenteral nutrition
SBS	Short bowel syndrome
